# Indents

**Published:** 1902-05-15

**Authors:** 


### INDENTS.

☞ **Brief Articles of Technical or Scientific Value will receive notice and comment. Contributions invited.**

TREATMENT OF ULCERATIVE STOMATITIS WITH CRYSTALS OF CHROMIC ACID.—M. Armand Siredey has achieved a cure in a number of cases of ulcerative stomatitis by cauterizing the diseased areas with crystals of pure chromic acid. He places the dry crystal upon the affected surface by means of a piece of sharpened stick. The cauterization is painful but easily bearable, and the pain does not last long. The cure is rapid and complete.

OXYGEN WITH ANESTHETICS FOR OLDER PEOPLE.—Greater allowance of oxygen should always be made for patients above middle age, although it often happens that their nervous and mental activity seems to make them more resistant to the action of the anesthetic. They should not, therefore, be heavily dosed with nitrous oxid to the extent of cyanosure. The general circulation at this time of life is less fitted to stand an asphyxial strain without the possibility of damage to the blood vessels or secondary cardiac depression.—*Journal of British Dental Association.*

CHLORETONE AS A PRESERVATION OF COCAIN SOLUTIONS.—Dr. Houghton, of Detroit, says he has kept cocain solutions for nearly a year by adding chloretone to the solutions, notwithstanding the fact that the bottle was frequently opened for use. The chloretone also assists the anesthetic property of the cocain. By experimentation on animals with cocain solutions to which chloretone has been added it is found that cocain phenomena are less liable to occur even with considerable doses. Less than one percent aqueous solutions of chloretone may be made.—*Dental Summary*.

GUAIACOL IN CAPPING PULPS.—Apply rubber dam, clean out soft debris and sterilize with a one to two hundred solution of bichlorid of mercury; then thoroughly dry the cavity for application of capping materials. Then make a suitable concave gold cap to fit cavity and embrace the exposed pulp. Incorporate in the guaiacol, sufficient cement powder to make a paste. Fill the gold cap with this paste and place it over the exposed pulp, and when set, flow thin cement over the cap to fill the cavity. Leave this in the tooth for several months and should no symptoms of pain appear, cut out a portion of the cement and replace it with gold.—C. P. Holland, in *Items of Interest*.

ARGENTAMIN IN PYORRHEA ALVEOLARIS.—Dr. W. B. Keyes in the January *Cosmos*, gives the following treatment for pyorrhea: First remove all deposits on the roots, and then with a flat, soft wood point saturated with aromatic sulphuric acid, rub the roots until smooth. Neutralize the acid with a suitable alkaline wash. Then apply a ten percent solution of argentamin, by means of a little cotton wound on a silver probe. Repeat this application at intervals according to indications, until the gums are healed. In the interval of treatments with the argentamin have the patient use a mouth wash containing salicylic acid and ratany. Argentamin is a solution of nitrate of silver in ethylendiamin and may be obtained of Schering & Glatz, N. Y. It is claimed to be a germicide of greater penetration and power than any other silver compound or even bichlorid of mercury. Care should be exercised that it be not applied so frequently as to produce cauterant reactions.

RULES FOR THE ADMINISTRATION OF COCAIN.—

(a) The dose of cocain to be injected should be proportionate to the extent of surface to be anesthetized; it should never be more than eight or ten centigrams. (Ten centigrams are one and one half grains, and is three times as much as should ever be hypodermically injected for dental operations. —Ed. REGISTER.)

(b) Cocain should never be used on persons suffering from cardiac affections, from chronic diseases of the respiratory tract, or from disorders of the nervous system.

(c) It should be injected *into* and not under the dermis of the mucous membranes or of the skin. This is the intradermic method of Professor Reclus in place of the hypodermic method. In this way the introduction of the substance within the veins, a condition that has occurred in several accidents recorded, will be avoided.

(d) The injection should always be performed with the patient in the horizontal position. Afterward if the operation is about the head the patient can be placed in the required position.

(e) The cocain should be absolutely pure. Certain mixtures of cocain with other alkaloids are of a particularly toxic nature.

(f) The quantity of cocain to be used in a single injection should be divided and a lapse ot a few minutes should be allowed between the first partial injection and the following. During this period it will be easy to observe if any toxic effects take place—for, as is well known, these will occur immediately after the first infection. This is the method of fractional injections.

(g) When used in this gradual and methodical way, cocain presents over the other general anesthetics (chloroform and ether) advantages upon which there is no occasion again to insist, and which merely need mention. Absence of general effects, of a period of excitement, of loss of consciousness, the possibility of operating without the aid of an assistant, as the dentist's intervention is consecutive to and not simultaneous with the introduction of the anesthetic agent.

(h) The duration of the anesthetic effect is always sufficient for the performance of all the operations of ordinary surgery.—*Journ. de Med, de Paris.*

[By "dermis of the mucous membrane" in paragraph (c) the author means the connective tissue corium which underlies the epithelial layer.—Ed. *Cosmos.*]

The most important rule in the use of cocain is that it should be well diluted. There is no occasion for using hypodermically a denser solution of cocain than one percent, for dental operations of any kind, except, obtunding dentin. Fifteen to thirty drops of a one percent solution of cocain can be safely used on most people without harm.—Ed. REGISTER.

